# Trends in Mortality Associated With Ischemic Heart Disease and Screenable Cancers in the United States, 1999 to 2024

**DOI:** 10.1016/j.jacadv.2025.102576

**Published:** 2026-01-19

**Authors:** Jamal S. Rana, Reed Mszar, Eugenia Gianos, Matthew J. Budoff, Khurram Nasir, Mushood Ahmed

**Affiliations:** a Department of Cardiology, The Permanente Medical Group, Oakland, California, USA; b Division of Research, Kaiser Permanente Northern California, Oakland, California, USA; c Department of Chronic Disease Epidemiology, Yale School of Public Health, New Haven, Connecticut, USA; d Northwell, Cardiovascular Institute, Lenox Hill Hospital, New York, New York, USA; e Northwell, Cardiovascular Institute, Peconic Bay Medical Centre, Riverhead, New York, USA; f Lundquist Institute at Harbor-UCLA Medical Centre, Los Angeles, California, USA; g Houston Methodist DeBakey Heart and Vascular Centre, Houston, Texas, USA; h Rawalpindi Medical University, Rawalpindi, Pakistan

**Keywords:** cancer, cardiovascular disease, prevention, screening, trends

Cardiovascular disease (CVD) and cancer are the 2 leading causes of morbidity and mortality in the United States. Significant improvements in pharmaceutical and interventional therapies have resulted in an overall decline in cardiovascular mortality during the past several decades. Yet the rate of these improvements has recently plateaued.^1^

On the other hand, mortality rates from certain cancer types (eg, breast, prostate, and colorectal cancer) with well-established and guideline-endorsed screenings (eg, mammograms, prostate-specific antigen tests, and colonoscopies) have declined considerably. Lung cancer deaths have also declined, with cancer screening and prevention efforts having averted nearly 4.8 million deaths over the past several decades.^2^ Despite earlier evidence that the gap between CVD and cancer mortality was closing in 2010, marked differences in their age-adjusted mortality rates (AAMRs) have persisted.^1^

We utilized the Centers for Disease Control and Prevention Wide-Ranging Online Data for Epidemiologic Research (CDC WONDER) to investigate contemporary trends in ischemic heart disease (IHD) mortality relative to these most prevalent screenable cancers.

## Methods

We analyzed mortality data from the National Vital Statistics System accessed via CDC WONDER, covering deaths in all 50 U.S. states and the District of Columbia from 1999 to 2024. Data were split into 2 periods: 1999 to 2011 and 2012 to 2024, as 2011 is considered a deflection point in cardiovascular mortality trends in the United States.^3^ Deaths were identified using International Classification of Diseases-10 codes for IHD (I20–I25), lung cancer (C34), breast cancer (C50), prostate cancer (C61), and colorectal cancer (C18–C20). The leading cancers with established routine screening guidelines were assessed alongside IHD for comparative trends. AAMRs per 100,000 were calculated among adults ≥25 years of age using the 2000 U.S. standard population. Joinpoint regression was applied to estimate average annual percent change (AAPC) over the study period. Provisional data through 2024 were included to ensure timely reporting. Institutional review board approval was not required, as data were publicly available and deidentified.

## Results

**Figure 1:**
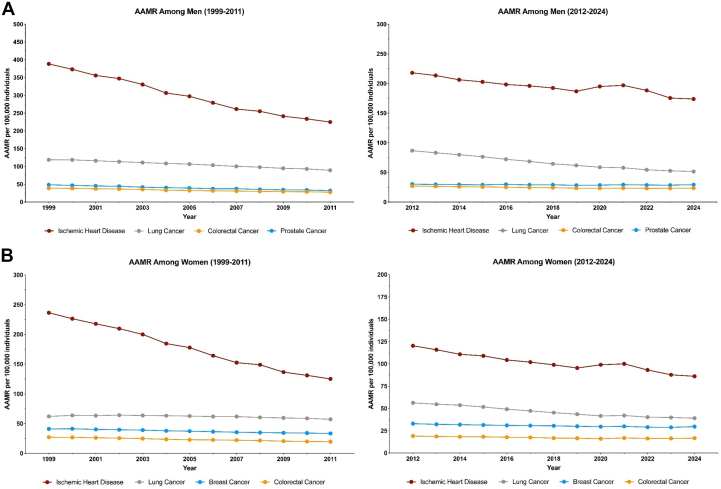
Trends in Mortality, Associated With Ischemic Heart Disease and Screenable Cancers in the United States, 1999-2024 (A) AAMRs among men for the ischemic heart disease and the leading cancer types with established screening guidelines, data from CDC WONDER (1999-2011 and 2012-2024). (B) AAMRs among women for the ischemic heart disease and the leading cancer types with established screening guidelines, data from CDC WONDER (1999-2011 and 2012-2024). AAMR = age-adjusted mortality rate; CDC WONDER = Centers for Disease Control and Prevention Wide-Ranging Online Data for Epidemiologic Research.

From 1999 to 2011 among men, all conditions exhibited significant declines in AAMRs (Figure 1). IHD demonstrated the steepest reduction (AAPC: −4.44%, 95% CI: −4.63% to −4.21%), followed by prostate cancer (−3.35%, 95% CI: −3.56% to −3.14%) and colorectal cancer (−2.74%, 95% CI: −2.90% to −2.51%). Lung cancer had a more modest decline (−2.33%, 95% CI: −2.46% to −2.21%). Between 2012 and 2024, lung cancer mortality demonstrated the most substantial reduction (AAPC: −4.37%, 95% CI: −4.68% to −4.15%). In contrast, the rate of decline for IHD slowed markedly (−1.95%, 95% CI: −2.24% to −1.73%). Declines in colorectal cancer were moderate in nature (−1.17%, 95% CI: −1.34% to −1.03%).

Among women from 1999 to 2011, IHD mortality declined sharply (AAPC: −5.26%, 95% CI: −5.84% to −4.89%), followed by colorectal cancer (−2.82%, 95% CI: −2.99% to −2.65%) and breast cancer (−1.87%, 95% CI: −2.02% to −1.72%). Lung cancer experienced a modest decline (−0.67%, 95% CI: −0.83% to −0.52%). From 2012 to 2024, lung cancer mortality among women saw a markedly accelerated rate of decline (AAPC: −2.96%, 95% CI: −3.15% to −2.78%). However, declines in IHD (−2.83%, 95% CI: −3.18% to −2.60%), colorectal cancer (−1.14%, 95% CI: −1.48% to −0.86%), and breast cancer (−0.87%, 95% CI: −1.05% to −0.71%) showed signs of plateauing during this more recent period.

## Discussion

In this analysis of population-level data from CDC WONDER, substantial reductions have been observed in the AAMRs attributable to IHD and multiple screenable cancers; however, the rate of these improvements has slowed considerably for IHD mortality in men and women with even a slight increase in mortality being observed during the COVID-19 pandemic. Of note, among men in 2024, the AAMR for IHD was more than 3-fold higher than that of lung cancer and higher than the rates for the 3 leading screenable cancers combined. Similarly, for women in 2024, the AAMR for IHD was nearly 2.2-times higher than that of lung cancer and equal to the combined rates of the 3 leading cancers. These findings highlight the persistent gap between IHD and cancer mortality, as well as the need for additional clinical and public health approaches to enhance current screening practices for IHD and further implement evidence-based strategies for averting preventable cardiovascular deaths.

In our analysis, the use of International Classification of Diseases codes and death certificates may have resulted in misclassification bias, and the database contains limited information including comorbidities, clinical laboratory data, or information on social determinants of health, which may serve as moderators and confounders for the observed trends. Specific screening strategies used to assess patients were also not available. Despite using JoinPoint regression in our analyses, we were unable to quantify the precise impact of the COVID-19 pandemic on the observed trends.

Similar to these cancer types, IHD meets the criteria for disease screening (ie, an identifiable predisease lesion, a positive response to treatment when detected early, and an extended incubation period). However, a major opportunity to systematize the use of coronary artery calcium (CAC) scanning in the appropriate patient is lacking. This is despite its relatively low cost (∼$100-150 vs ∼$3,000 for a colonoscopy), ease of administration and interpretation, and low radiation exposure (equivalent to ∼1 mSv mammogram).^4^ While observational data show the power of CAC for risk prediction, and clinical trial data show that randomization to CAC scanning is associated with superior CVD factor control, CAC screening remains significantly underutilized, particularly in underserved communities. So, where can we move the needle? Increased education of clinicians to impact clinical inertia with more widespread CAC screening is needed, along with legislative reform to provide insurance coverage beyond only 3 states of Texas, New Mexico, and Connecticut that have enacted mandates requiring insurance coverage.

With a plethora of preventive therapies showing the benefit of early aggressive lipid lowering, the ability to impact global mortality with early screening is tremendous. An entry criterion for VESALIUS-CV (Effect of Evolocumab in Patients at High Cardiovascular Risk Without Prior Myocardial Infarction or Stroke) trial was CAC ≥100, with evidence of 25% risk reduction with evolocumab.^5^ Further, 19 to 20 million noncardiac chest CT scans, including those for lung cancer screening done annually in the United States, afford an opportunity to measure CAC and implement therapeutics accordingly to prevent future IHD.

## Funding support and author disclosures

The authors have reported that they have no relationships relevant to the contents of this paper to disclose.
